# Supplementary material for: Genome Mining and Genetic Manipulation Reveal New Isofuranonaphthoquinones in Nocardia Species
Source: Int J Mol Sci. 2024 Aug 14;25(16):8847. doi: 10.3390/ijms25168847 (PMC11354674; doi:10.3390/ijms25168847)
Supplement: Supplementary file 1 [file ijms-25-08847-s001.zip › ijms-3144892-supplementary.pdf]

International Journal of
Molecular Sciences

MDPI

Genome Mining and Genetic Manipulation Reveal New Isofuranonaphthoquinones in Nocardia Species

Purna Bahadur Poudel [1,†], Dipesh Dhakal [1,†], Rubin Thapa Magar [1], Niranjan Parajuli [1,2] and Jae Kyung Sohng [1,3,*]

Table S1. Predicted functions of genes in the *fnq* gene cluster.

Protein	Size (AA)	Proposed function	Closest homologs and origin	I/S (%)	(Guo *et al*., 2016)	(Katsuyama *et al*., 2016)
Orf1	245	NAD(P)H nitroreductase	WP_199856738.1, *Nocardia suismassiliense*	94/96	-	-
Orf2	579	GAF domain-containing sensor histidine kinase	WP_194832974.1, *[Nocardia sp.* XZ_19_369	95/96	-	-
FnqA	376	Esterase	GEM36072.1, *Nocardia ninae* NBRC 108245	94/96	IfqF (APR73630)	IfnF (BAU98033)
FnqB	62	Ferredoxin	WP_147128374.1, *Nocardia ninae*	95/96	-	-
FnqC	406	cytochrome P450	WP_107657096.1, *Nocardia suismassiliense*	93/96	-	-
FnqD	160	Flavin reductase	WP_107657097.1, *Nocardia suismassiliense*	98/99	IfqP (APR73639)	IfnP (BAU98043)
FnqE1	82	Acyl-carrier protein (ACP)	WP_222594955.1, *Nocardia ninae*	99/100	IfqA (APR73625)	IfnA (BAU98028)
FnqE2	430	PKS II-KSα	GEM36067.1, *Nocardia ninae*	98/98	IfqN (APR73637)	IfnN (BAU98041)
FnqE3	422	PKSII-KSβ	WP_194832970.1, *Nocardia sp.* XZ_19_369	93/96	IfqO (APR73638)	IfnO (BAU98042)
FnqF	422	LuxR C-terminal-related transcriptional regulator	WP_194832969.1, *Nocardia sp.* XZ_19_369	99/99	IfqM (APR73636)	IfnM (BAU98040)
FnqG	141	SnoaL-like domain protein	WP_147128368.1, *Nocardia ninae*	97/98	IfqT (APR73643)	IfnT (BAU98047)
FnqH	501	FAD oxidase	WP_199856740.1, *Nocardia suismassiliense*	97/99	-	-

FnqI	605	Bayer-Villiger monoxygenaseE	WP_222594953.1, *Nocardia ninae*	95/97	IfqQ (APR73640)	IfnQ (BAU98044)
FnqJ	388	Hydrolase	WP_147128366.1, *Nocardia ninae*	98/99	IfqR (APR73641)	IfnR (BAU98045)
FnqK	581	acetyl-CoA carboxylase, carboxyltransferase subunit beta	WP_147128365.1, *Nocardia ninae*	97/99	-	-
FnqL	454	acetyl-CoA carboxylase biotin carboxylase subunit	WP_147128364.1, *Nocardia ninae*	99/99	-	-
FnqM	129	lipocalin-like domain-containing protein	WP_147128363.1, *Nocardia ninae*	95/97	IfqL (APR73635)	IfnL (BAU98039)
FnqN/NgnL	308	Alpha/beta hydrolase	WP_147128362.1, *Nocardia ninae*	97/98	-	-
FnqO	251	AfsR/SARP family transcriptional regulator	WP_194832961.1, *Nocardia* sp. XZ_19_369	99/99	IfqD (APR73628)	IfnD (BAU98031)
FnqP	128	Helix-turn-helix transcriptional regulator	WP_147128360.1, *Nocardia ninae*	95/96	-	-
FnqQ	280	aromatase/cyclase	WP_147128359.1, *Nocardia ninae*	99/99	IfqJ (APR73633)	IfnJ (BAU98037)
FnqR	249	Cyclase family protein	WP_147128358.1, *Nocardia ninae*	99/99	IfqK (APR73634)	IfnK (BAU98038)
FnqS	181	acetyl-CoA carboxylase biotin carboxyl carrier protein subunit	WP_147128357.1, *Nocardia ninae*	94/96	-	-
Orf3	394	GGDEF domain-containing protein	WP_147128356.1, *Nocardia ninae*	94/96	-	-
Orf4	477	M14 family zinc carboxypeptidase	WP_147128355.1, *Nocardia ninae*	96/97		
ThnM3	347	Methyltransferase	WP_211269074.1, *Saccharothrix syringae*	65/75		

Table S2. Production level of NOC-IBR1 and NOC-IBR2 in different strains of *Nocardia*. The production level was reported as average from 3 readings with respective standard deviation

**Bacterial strains**	**Description**	**Production titer (mg/L)**	
		NOC-IBR1	NOC-IBR2
Nocardia sp. CS682	Wild-type producer of nargenicin A1	0.03	0.16
Nocardia sp. CS682/fnqO	Wild strain with overexpression of SARP gene	0.09	0.56
Nocardia sp. CS682DR	Mutant strain with inactivation of nargenicin biosynthesis	0	0.46
Nocardia sp. CS682DR/fnqO	Mutant strain with overexpression of SARP gene	0.821	2.782
Nocardia sp. CS682DR ΔfnqO	Mutant strain with inactivation of SARP gene	0	0
Nocardia sp. CS682DR ΔfnqO/p18fnqO	Complementation strain *Nocardia* CS682DR ΔfnqO	0	2.61

Table S3. Antibacterial activities test against Gram-positive and Gram-negative bacteria via disk diffusion assay. The zone of inhibition (diameter) due to NOC-IBR1, NOC-IBR2, erythromycin, and methanol against different pathogens is noted in mm

	NOC-IBR1	NOC-IBR2	Erythromycin	Methanol
**Gram-Positive Strains**				
S. aureus CCARM 3640 (MRSA)	ND	32 ± 0.2	ND	ND
S. aureus CCARM 3634 (MRSA)	ND	29 ± 0.15	ND	ND
S. aureus CCARM 33591(MRSA)	ND	25 ± 0.25	ND	ND
S. aureus CCARM 0204 (MSSA)	ND	20 ± 0.28	30 ± 0.28	ND
S. aureus CCARM 3090 (MRSA)	ND	18 ± 0.35	10 ± 0.19	ND
S. aureus CCARM 3635 (MRSA)	ND	18 ± 0.25	ND	ND
S. aureus CCARM 3089 (MRSA)	ND	15 ± 0.29	ND	ND
S. aureus CCARM 0205 (MSSA)	ND	14 ± 0.21	33 ± 0.28	ND
S. aureus CCARM 0027 (MSSA)	ND	10 ± 0.2	31 ± 0.28	ND
Bacillus subtilis ATCC 6633	ND	ND	ND	ND
Enterococcus faecalis 19433	ND	ND	ND	ND
Enterococcus faecalis 19434	ND	ND	ND	ND
Kocuria rhizophilla NBRC 12708	ND	ND	ND	ND
Micrococcus luteus	ND	ND	ND	ND
**Gram-Negative Strains**				
Escherichia coli ATCC 25922	ND	ND	ND	ND
Proteus hauseri NBRC 3851	ND	ND	ND	ND
Klebsiella pneumonia ATCC10031	ND	ND	ND	ND
Salmonella enterica ATCC 14028	ND	ND	ND	ND

Table S4. Anticancer (IC_{50} (μM)) potential of NOC-IBR1, NOC-IBR2 and Doxorubicin against different cell lines

**Cell Lines**	**NOC-IBR1**	**NOC-IBR2**	**Doxorubicin**
**Cancer cell lines**			
**A549**	199.5	112.5	0.103
**Huh7**	44.44	54.18	0.193
**HeLa**	61.14	29.27	0.071
**U87MG**	119.6	118.5	0.105
**Normal cell lines**			
**HaCaT**	>200	>200	0.681

Table S5. The kinetic parameter of ThnM3 with NOC-IBR1 and *S*-adenosyl-L-methionine.

**Substrate**	**K_m (μM)**	**V_{max} (μM min^{-1}μg^{-1})**
NOC-IBR1	15.62 ± 1.75	0.048 ± 0.002
S-adenosyl-L-methionine	39.7 ± 6.08	0.021 ± 0.002

Table S6. List of strains and plasmids used in this study for heterologous expression and constructed for mutant strain

Bacterial strains	Description	Source/reference
E. coli XL1Blue	*Δ(mcrA)183 Δ(mcrCB-hsdSMR-mrr)173 endA1 supE44 thi-1 recA1 gyrA1 gyrA96 relA1 lac*	Stratagene
E. coli ET-12567	DNA demethylating strain (*dam dcm hsdS* cm^R)	John Innes Center, UK
E. coli BL21(DE3)	B; F- *omp*T *hsd*SB (*rB-mB-*) *gal dcm* (DE3)	Invitrogen
E. coli-thnM3	*E. coli* containing pET32-*thnM3*	This study
E. coli- narM	*E. coli* containing pET32-*narM*	(Dhakal et al., 2020)
Nocardia sp. CS682	Wild-type producer of nargenicin A1	(Sohng et al., 2008)
Nocardia sp. CS682DR	Mutant strain with inactivation of nargenicin biosynthesis	(Mishra et al., 2019)
Nocardia sp. CS682DR/fnqO	Mutant strain with overexpression of SARP gene	This study
Nocardia sp. CS682DR ΔfnqO	Mutant strain with inactivation of SARP gene	This study
Nocardia sp. CS682DR ΔfnqO/p18fnqO	Complementation strain *Nocardia* CS682DR ΔfnqO	This study
**Plasmids and rDNAs**		
pGEM-T®-easy vector	*E. coli* general cloning vector, Amp^r	Promega, Madison, WI, USA
pCRISPomyces-2	Streptomyces expression of codon-optimized Cas9 and custom gRNA	Addgene, Watertown, MA, USA
pET32a(+)	Single T7 promotor, pBR322 *ori*, Km^r	Novagen
pET32-*thnM3*	pET32a(+) containing *thnM3* methyltransferase	This study
pET32a(+)-*narM*	pET32a(+) containing *narM* methyltransferase	(Dhakal et al., 2020)
pNV18L2	*Nocardia-E. coli* shuttle vector, neo^r	(Dhakal et al., 2016)
p18fnqO	pNV18L2 for expression of SARP for complementation	This study.

Table S7. List of oligonucleotides used in this study for gene cloning

Gene	Sequence (5′-3′)	RS	Notes
Del-fnqO - up-F	TCTAGAATGGCTCGTCAGCCC GCCTAC	*Xba*I	For amplification of upstream region for deletion of SARP
Del--fnqO- up-R	GGATCCCCCGGGGTGTCGGA CATGCGTC	*Bam*HI	
Del--fnqO - dn-F	GGATCCAGCGATCCCGCCGG GCTTTAC	*Bam*HI	For amplification of downstream region for deletion of SARP
Del--fnqO - dn-F	ACTAGTTGCTGCGGTCCCGGC CAATTC	*Spe*I	
Ove-*fnqO-F*	GGATCCATGAGGTTCAAGCTG CTCGGC	*Bam*HI	For overexpression of the SARP regulator gene, *fnqO*
Ove-*fnqO-R*	GGTACCTTAATAAGCCACCCG GTCCTC	*Kpn*I	
fnqO fragment	ATGAGGTTCAAGCTGCTCGGC CAGTTCGAAATCGTCGCCGAC GAAGGGCCGATCCTGTTGAC ACAATCGAAAATAAGCCAAC TACTCGGCTTGTTGTTGATTCA GAACGGCGAGACGGTGAGCG TGGATTCCTTGATCGAGGAAC TCTGGGGCGAGGACATGCCC CGCAGCGCGCTCACCACCCTG CAAACCTACGTCTACCACGCC CGCAAGATGTTCGCGGCGCTG TCCGGCGGCAAGGACATCCT GGTCACCAGGCCGTCCGGGT ACGCGATCGAGGTGGCCGAC GAGTCGGTCGACGTGCGGGC ATACATCGCGCACGTCGACA AGGCCACAAAAGCGTACGCA CTCGGTGATGTGGAATCGGTC ATCGATCATTTGGAGGTGACC CGCAAATTGTGGCGCGGGCC GTTTCTGGTCGGCATTCCGAA GGGCCAGGTACTCGACGCCT ACGTCACCTATCTGAAAGAA GTACGACTCACGGCGCTCGA ACTAGACATCGAAATCAAGC AGCGGACAGGCAATTACCGA GGACTTATTCCGCAACTTCGG ***CTT***CTGGTGGCCGAGAATCCG CTCAACGAAAATCTGCATGCC CAATTGATAAAGGTGCTGCAC AAGTGCGGGCGGCGGGCGGA		For insertion in the the *Bam*HI and *Kpn*I sites of pNV18L2 to generate pNV18L2-*fnqO*

	GGCGCTGGCGGCGTATCGCG<br>ATCTGTGGCAGGTACTCGACG<br>CGGAATTGGGTGTGCGGCCG<br>ACCTCGGAACTCCAGTCGATT<br>CAGCAGGAACTGCTGACCGA<br>GGACCGGGTGGCTTATTAA		
guide RNA	GAGACATCTTTGAAGACAAac<br>gcTCGAAAATAAGCCAACTAC<br>Tgttttagagctagaaatagcaagttaaaata<br>aggctagtccgttatcaacttgaaaaagtggc<br>accgagtcggtgctttttttagcataaccccttgg<br>ggcctctaaacgggtcttgaggggtttttttggc<br>tgctccttcggtcggacgtgcgtctacgggcac<br>cttaccgcagccgtcggctgtgcgacacggac<br>ggatcgggcgaactggccgatgctgggaga<br>agcgcgctgctgtacggcgcgcaccgggtgc<br>ggagcccctcggcgagcggtgtgaaacttctg<br>tgaatggcctgttcggttgctttttttatacggct<br>gccagataaggcttgcagcatctgggcggcta<br>ccgctatgatcggggcgttcctgcaattcttagt<br>gcgagtatctgaaaggggatacgcTCTGC<br>ATGCCCAATTGATAA g<br>tttAAGTCTTCTTTCACGTGGC		For insertion in the *Bbs*I site of pCRISPomyces-2

Region	Type	From	To	Most similar known cluster	
Region 1	NRPS-like	51,092	94,598		
Region 2	lanthipeptide-class-i	374,822	399,258	calicheamicin	Polyketide
Region 3	CDPS	405,665	426,387		
Region 4	redox-cofactor	437,574	459,627	lankacidin C	NRP+Polyketide
Region 5	NRPS, T1PKS	478,939	566,643	foxicin A/foxicin B/foxicin C/foxicin	NRP+Polyketide
Region 6	butyrolactone	588,576	599,589		
Region 7	T1PKS	609,088	693,486	butyrolactol A	Polyketide
Region 8	T2PKS, PKS-like, T1PKS	770,703	906,587	nargenicin	Polyketide
Region 9	LAP	959,258	983,834	ketomemicin B3/ketomemicin B4	Other
Region 10	T1PKS	1,037,668	1,083,586	calicheamicin	Polyketide
Region 11	T3PKS, terpene	1,093,394	1,134,617	merochlorin A/merochlorin B/deschloro-merochlorin A/deschloro-merochlorin B/isochloro-merochlorin B/dichloro-merochlorin B/merochlorin D/merochlorin C	Terpene+Polyketide:Type III polyketide
Region 12	ectoine	1,212,677	1,223,075	ectoine	Other
Region 13	NRPS	1,254,653	1,308,473	calicheamicin	Polyketide
Region 14	NRPS-like	1,392,796	1,435,408		
Region 15	terpene	1,538,468	1,559,580		
Region 16	other, phosphonate	1,811,113	1,851,985	phosphinothricintripeptide	NRP
Region 17	NRPS	2,457,387	2,510,844		
Region 18	NRPS	2,633,051	2,680,103	heterobactin A/heterobactin S2	NRP
Region 19	T1PKS	2,714,510	2,801,895	triacsin C	Other
Region 20	NRPS	2,881,420	2,925,814	diisonitrile antibiotic SF2768	NRP
Region 21	NRPS	2,964,778	3,009,034	clipibicyclene/azabicyclene B/azabicyclene C/azabicyclene D	NRP+Polyketide
Region 22	lanthipeptide-class-iii	3,377,072	3,399,588		
Region 23	aminopolycarboxylic-acid	3,496,242	3,509,665	EDHA	Other
Region 24	RiPP-like, NRPS	3,660,978	3,749,547	corynecin III/corynecin I/corynecin II	Other
Region 25	ranthipeptide	3,763,669	3,785,222	incednine	Polyketide
Region 26	RiPP-like	4,405,909	4,416,781		
Region 27	T1PKS	4,503,541	4,548,124		
Region 28	T1PKS	4,787,469	4,832,916		
Region 29	NAPAA	5,070,820	5,104,737	ε-Poly-L-lysine	NRP
Region 30	NRP-metallophore, NRPS	5,357,580	5,404,465	mycobactin	NRP+Polyketide
Region 31	NRPS, terpene, NRP-metallophore, lanthipeptide-class-iii	5,482,450	5,620,635	nocobactin NA/nocobactin NA 10152B	NRP+Polyketide
Region 32	NRPS	6,225,981	6,273,048		
Region 33	CDPS	6,321,409	6,342,152		
Region 34	butyrolactone	6,620,422	6,631,414	cyphomycin	Polyketide
Region 35	terpene	7,054,283	7,075,428	isorenieratene	Terpene
Region 36	aminopolycarboxylic-acid, arylpolyene	7,275,766	7,321,751	[S,S]-EDDS	Other
Region 37	NRPS	7,544,811	7,590,993		
Region 38	terpene, NRPS-like	7,606,637	7,657,576	geosmin	Terpene
Region 39	lanthipeptide-class-iv, NRPS-like, PKS-like	7,750,385	7,809,966	bonnevillamide D/bonnevillamide E	NRP
Region 40	NRPS-like, NRPS	7,887,772	7,933,934	anthramycin	NRP
Region 41	terpene	7,967,169	7,988,158	isorenieratene	Terpene
Region 42	lassopeptide	8,023,168	8,045,676	calicheamicin	Polyketide
Region 43	T3PKS	8,121,235	8,162,359	thiolutin	NRP
Region 44	hglE-KS	8,344,792	8,391,196	polyketomycin	Polyketide:Iterative type I polyketide+Polyketide:Type II polyketide+Saccharide:Hybrid/ta saccharide
Region 45	T1PKS, NRPS	8,437,751	8,494,647		
Region 46	other	8,497,937	8,538,839	tetrocarcin A	Polyketide

Figure S1. Identification of secondary metabolites regions of *Nocardia sp*. CS682 using antiSMASH 7.1.0 (March 15, 2024).

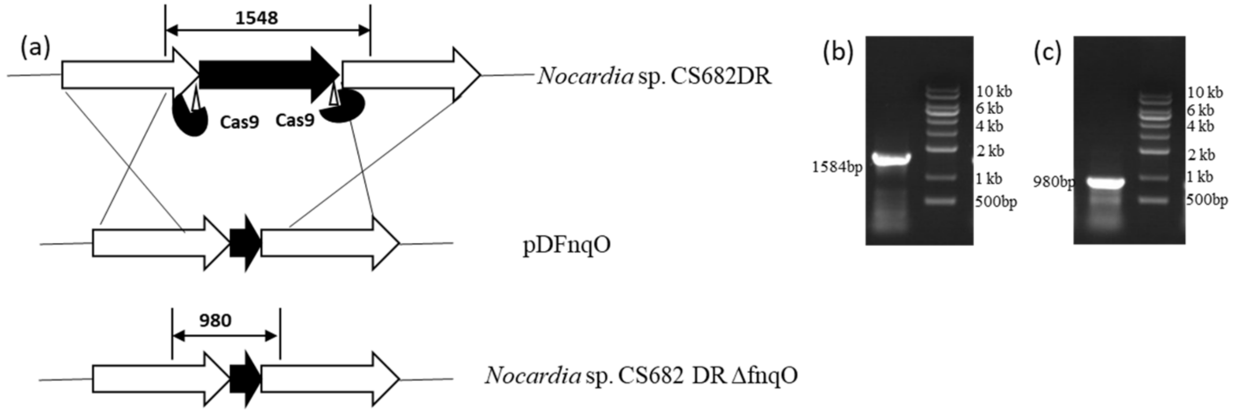

Figure S2. Construction of *fnqO* deletion mutant, *Nocardia* sp. CS682DR ΔfnqO (a) Depiction of *Nocardia* sp. CS682DR ΔfnqO.Gel electrophoresis of PCR products. DNA templates were from (b) *Nocardia* sp. CS682DR, and (c) deletion mutant *Nocardia* sp. CS682DR ΔfnqO.

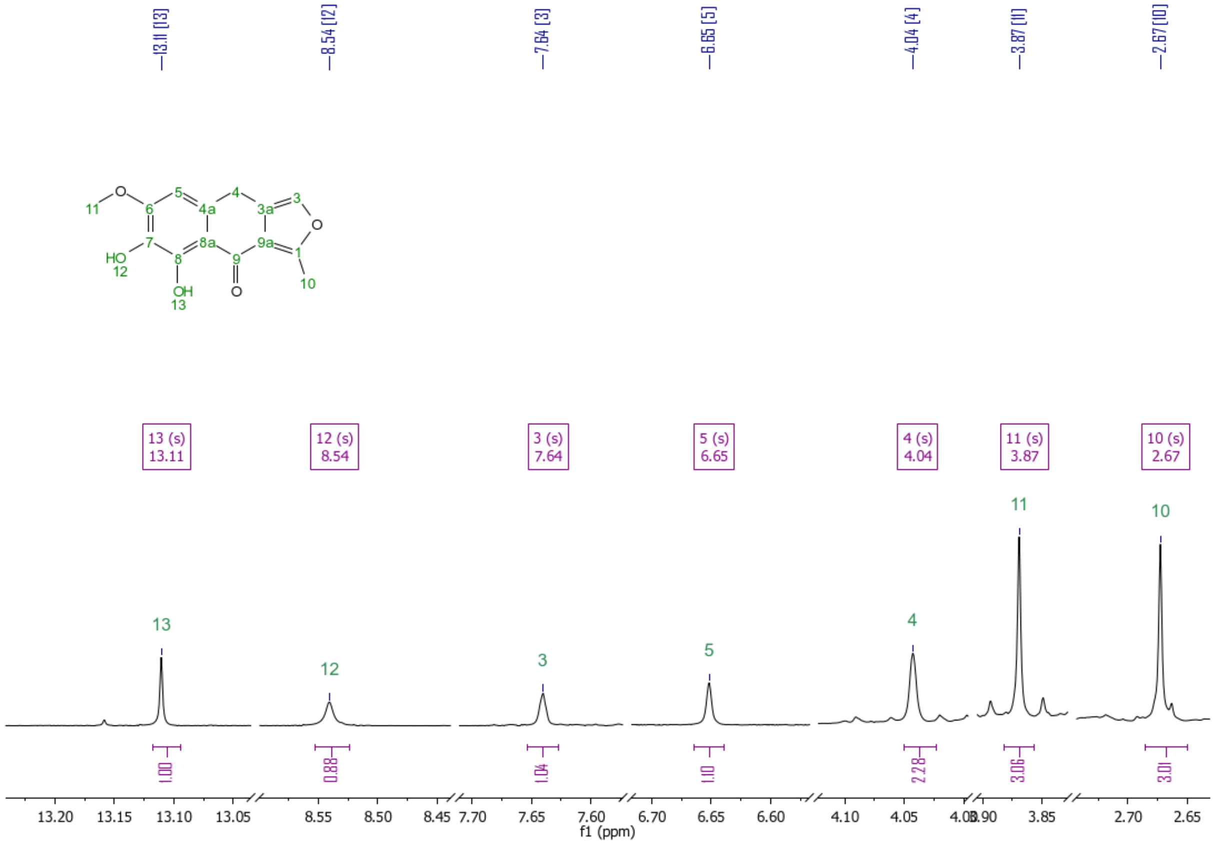

Figure S3.

(a) ^{1}H NMR spectrum of NOC-IBR1 in 700MHz, DMSO-d6

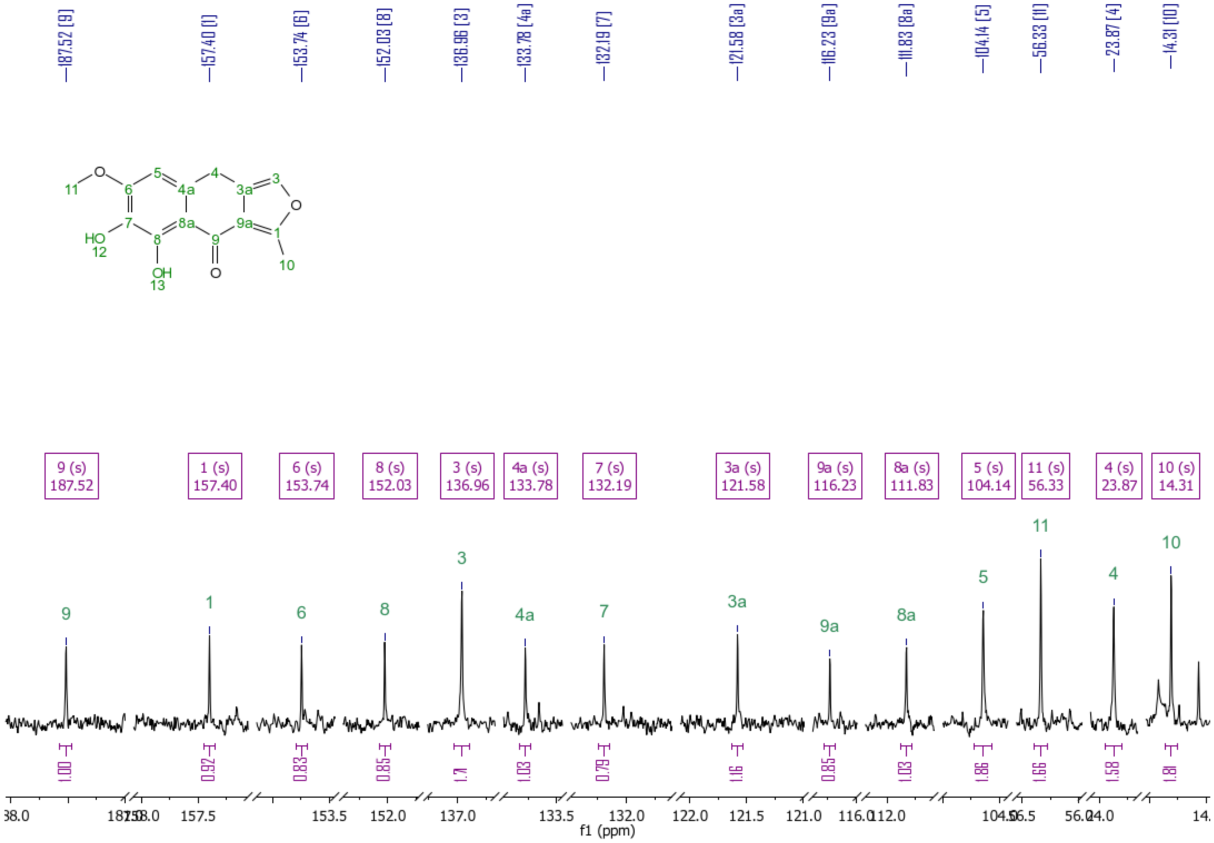

(b) ^{13}C NMR spectrum of NOC-IBR1 in 176 MHz, DMSO-d6

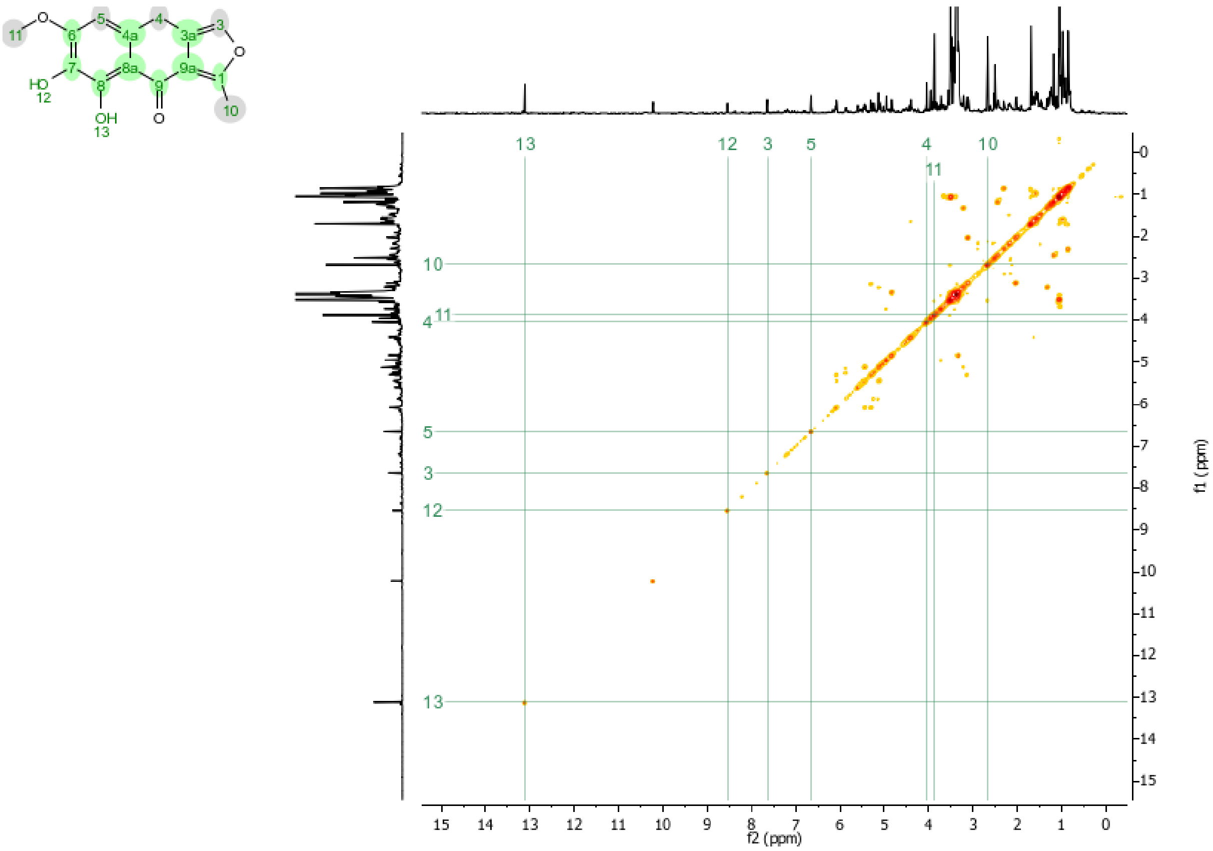

(c) ^{1}H-^{1}H COSY NMR of NOC-IBR1

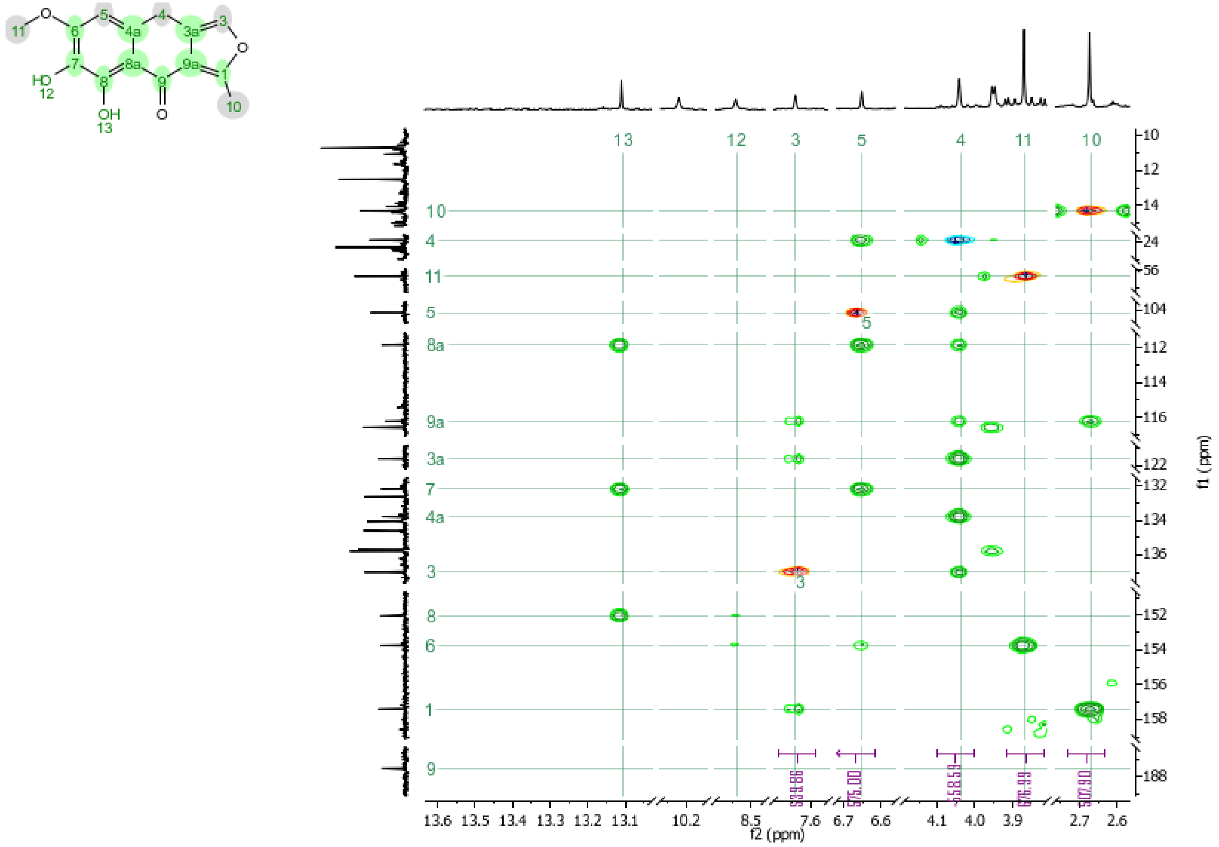

(d) ^{1}H-^{13}C HSQC-DEPT NMR of NOC-IBR1

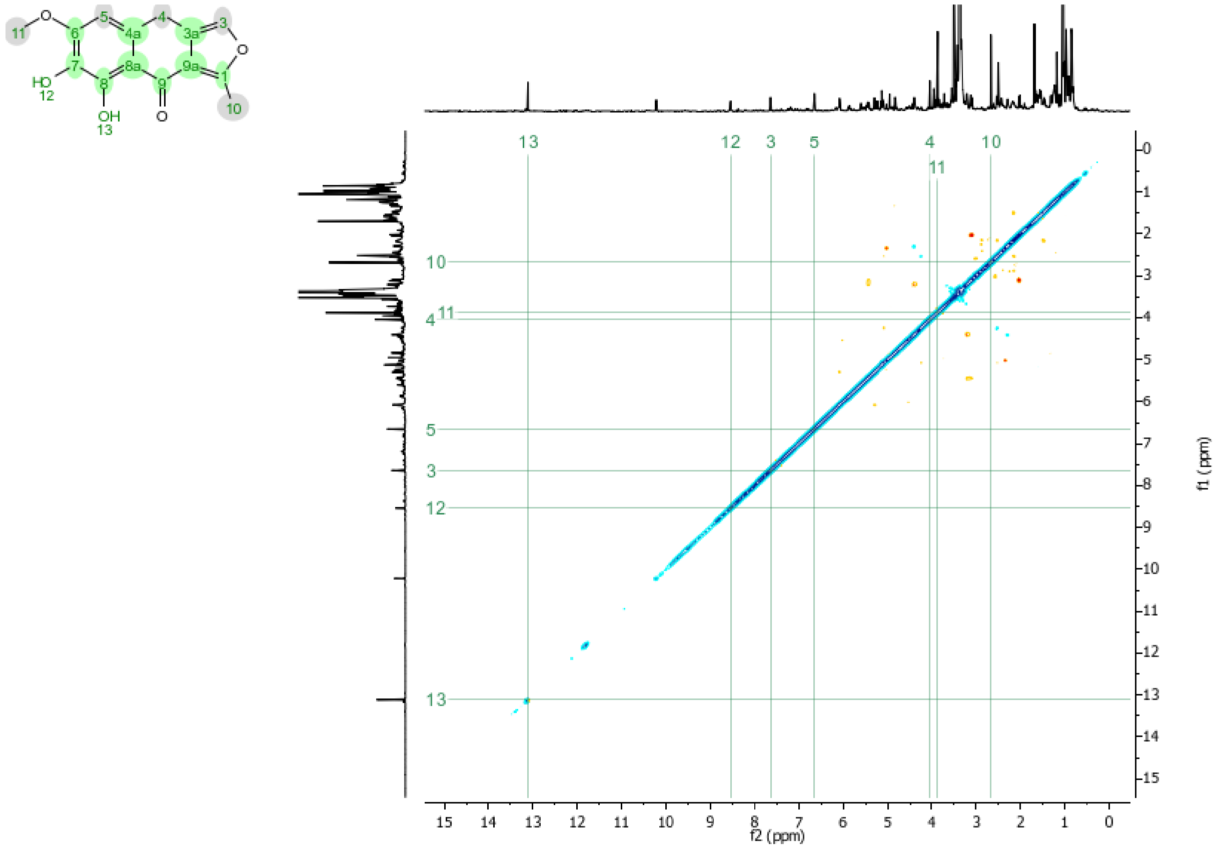

(e) ^{1}H-^{1}H ROSEY NMR of NOC-IBR1

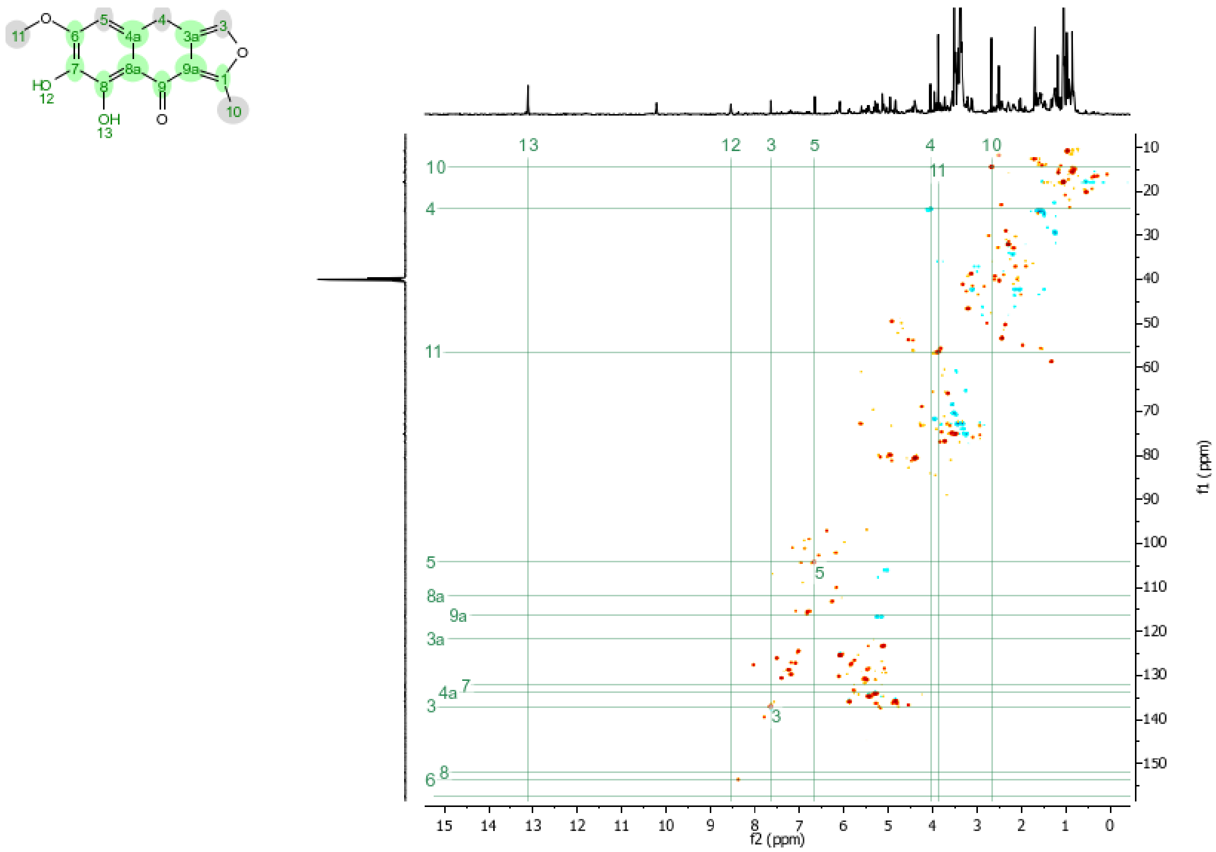

(f) ^{1}H-^{13}C HSQC-DEPT NMR of NOC-IBR1

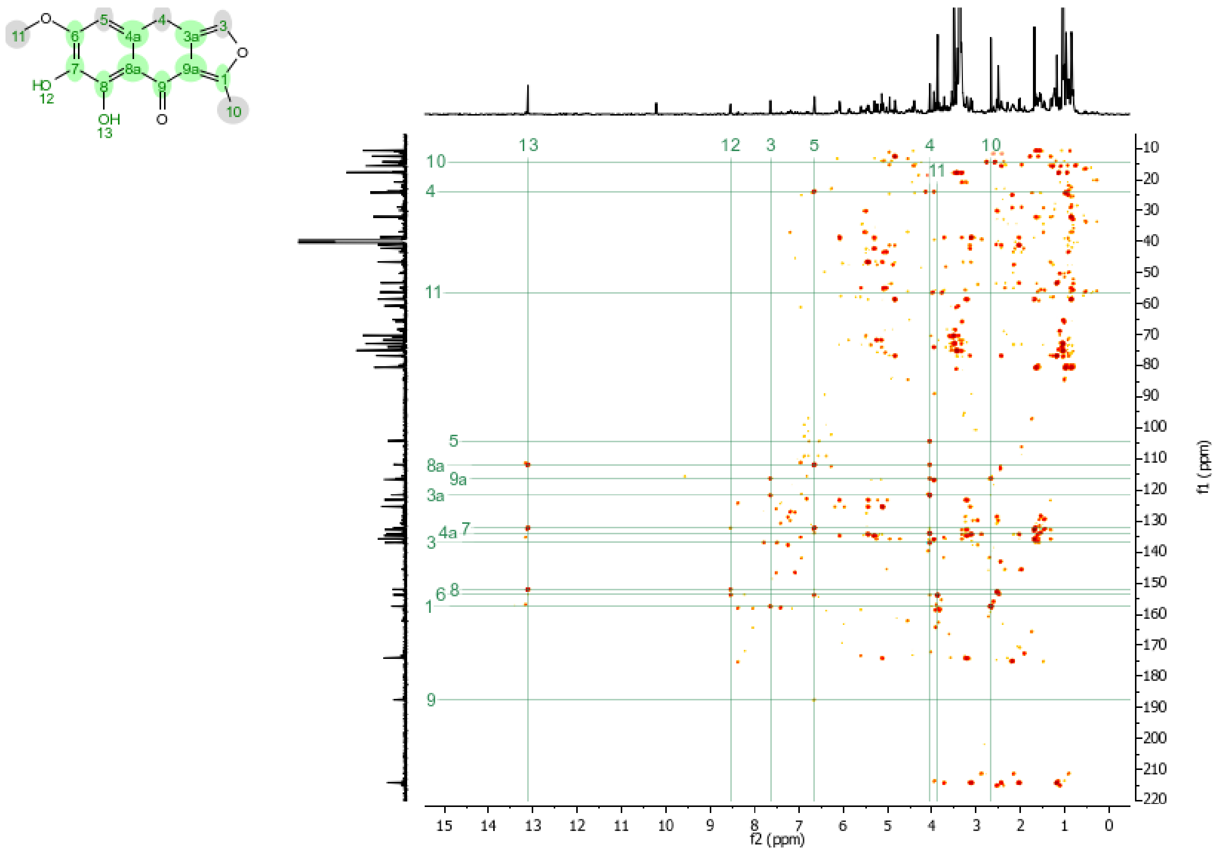

(g) ^{1}H-^{13}C HMBC NMR of NOC-IBR1

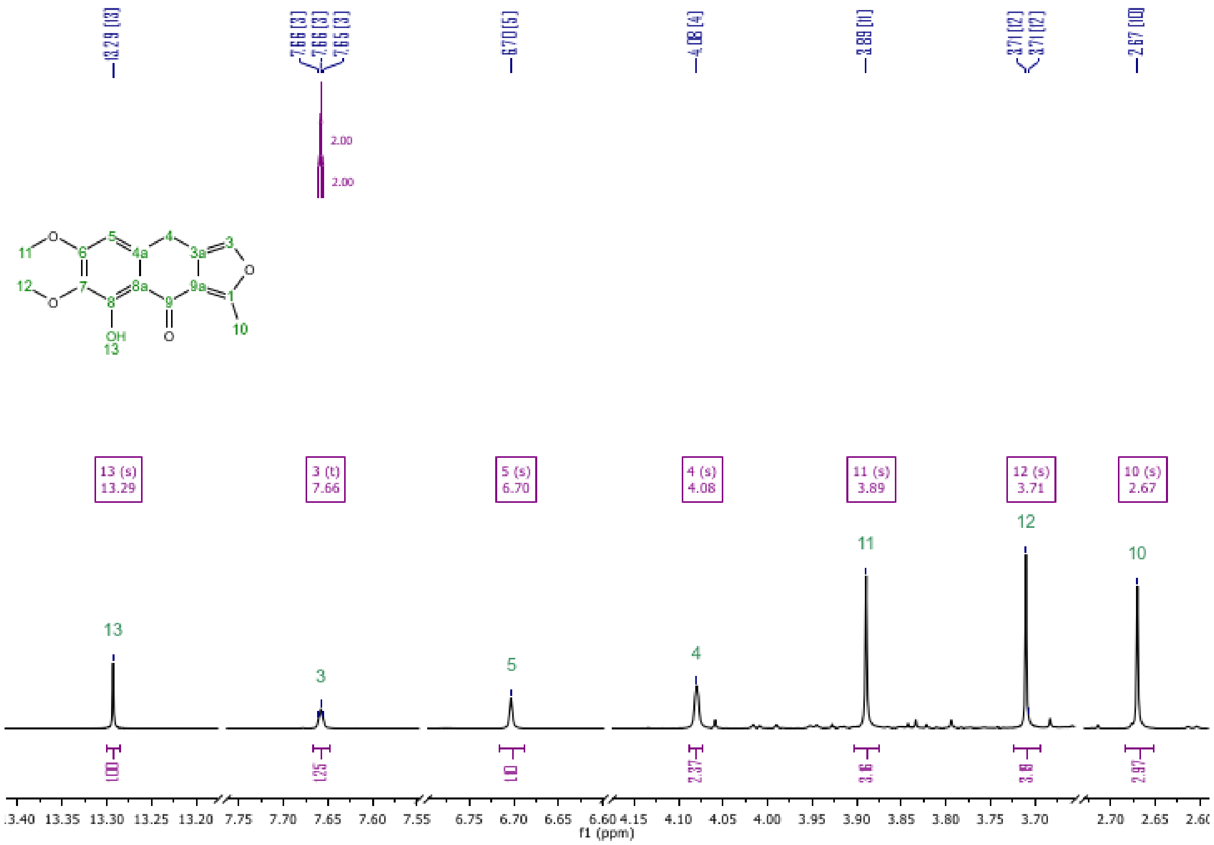

Supplementary Figure S4.

(a) ^{1}H NMR spectrum of NOC-IBR2 in 700MHz, DMSO-d6

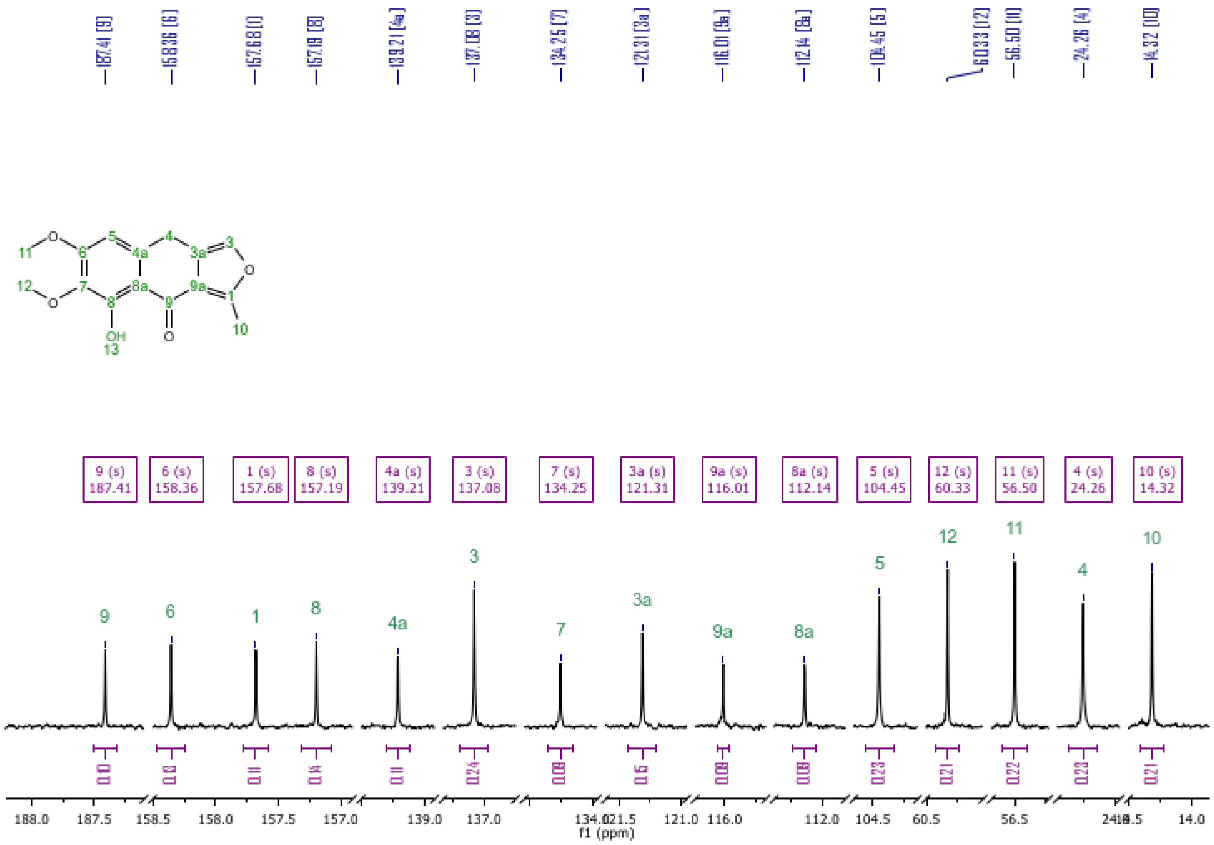

(b) ^{13}C NMR spectrum of NOC-IBR2 in 176 MHz, DMSO-d6

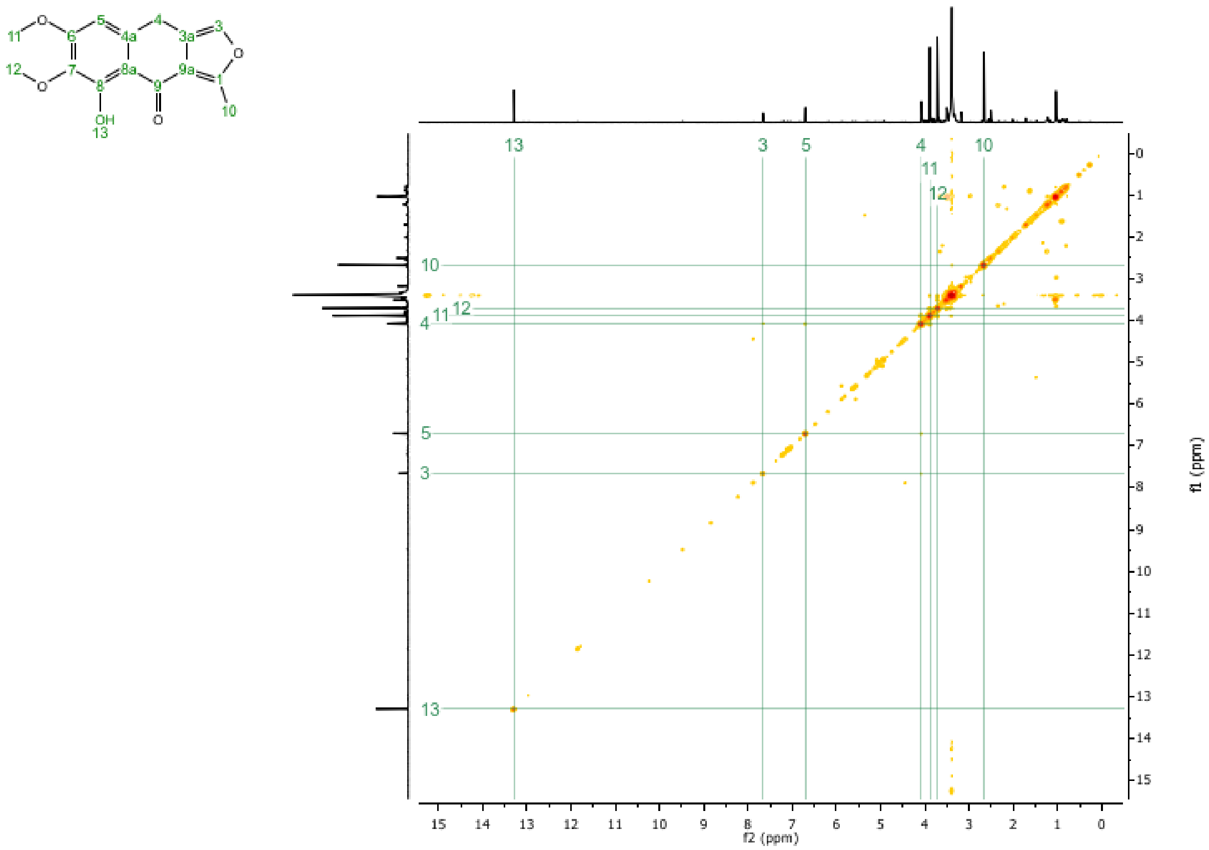

(c) ^{1}H-^{1}H COSY NMR of NOC-IBR2

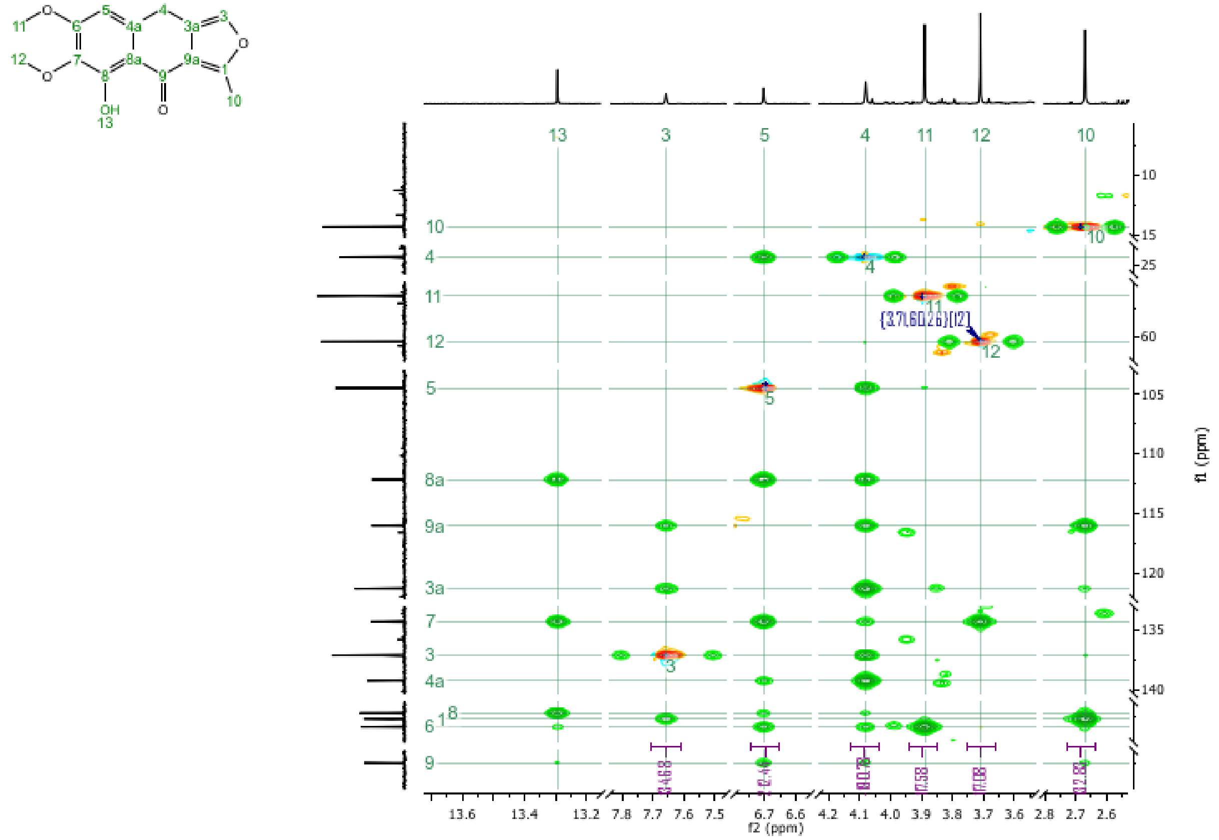

(d) ^{1}H-^{13}C HSQC-DEPT NMR of NOC-IBR2

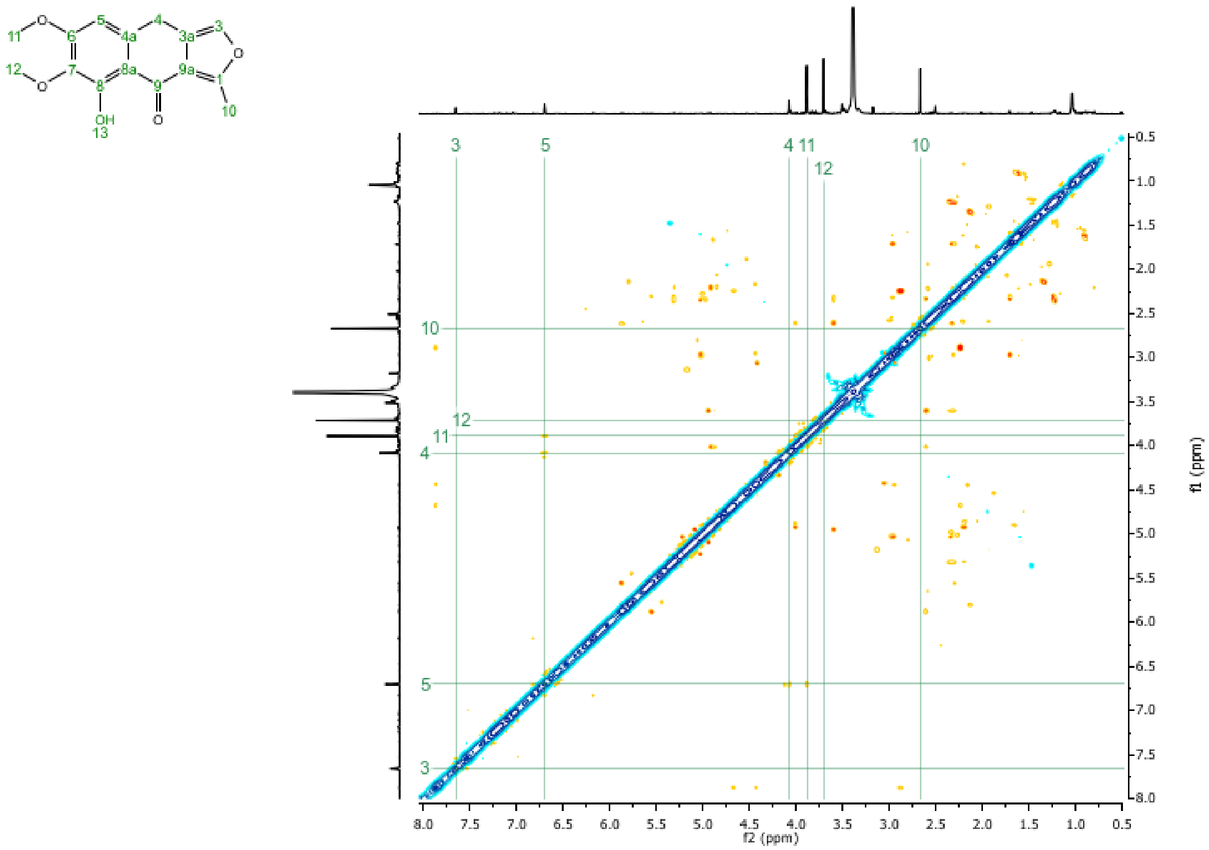

(e) ^{1}H-^{1}H ROSEY NMR of NOC-IBR2

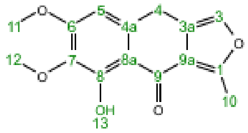

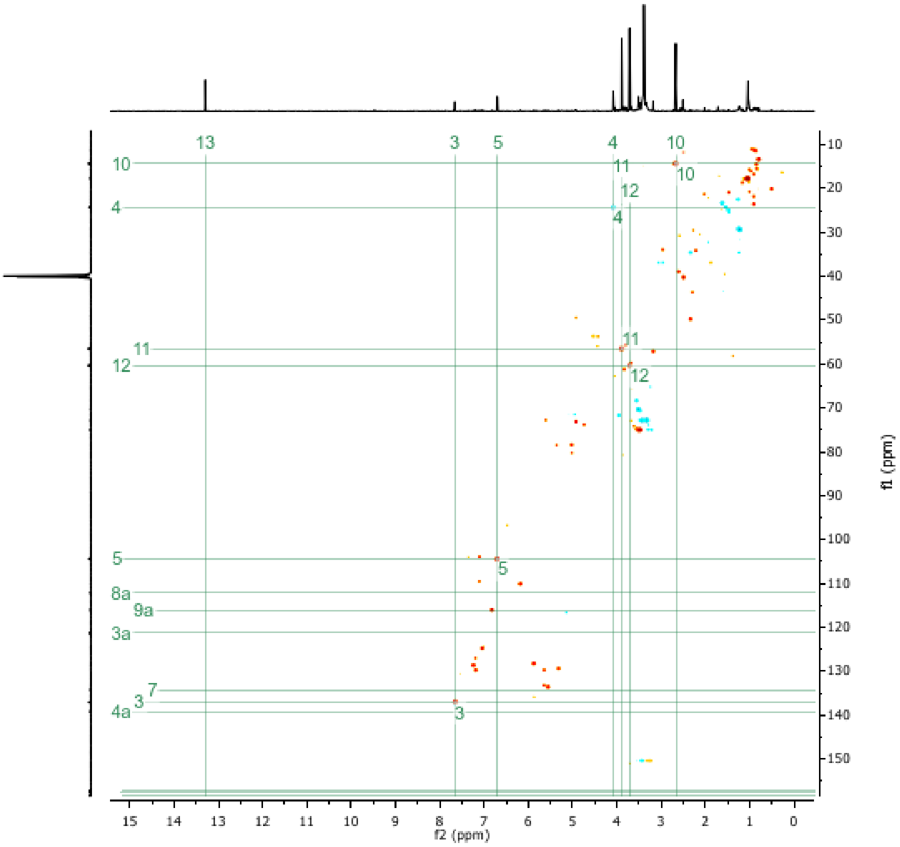

(f) ^{1}H-^{13}C HSQC-DEPT NMR of NOC-IBR2

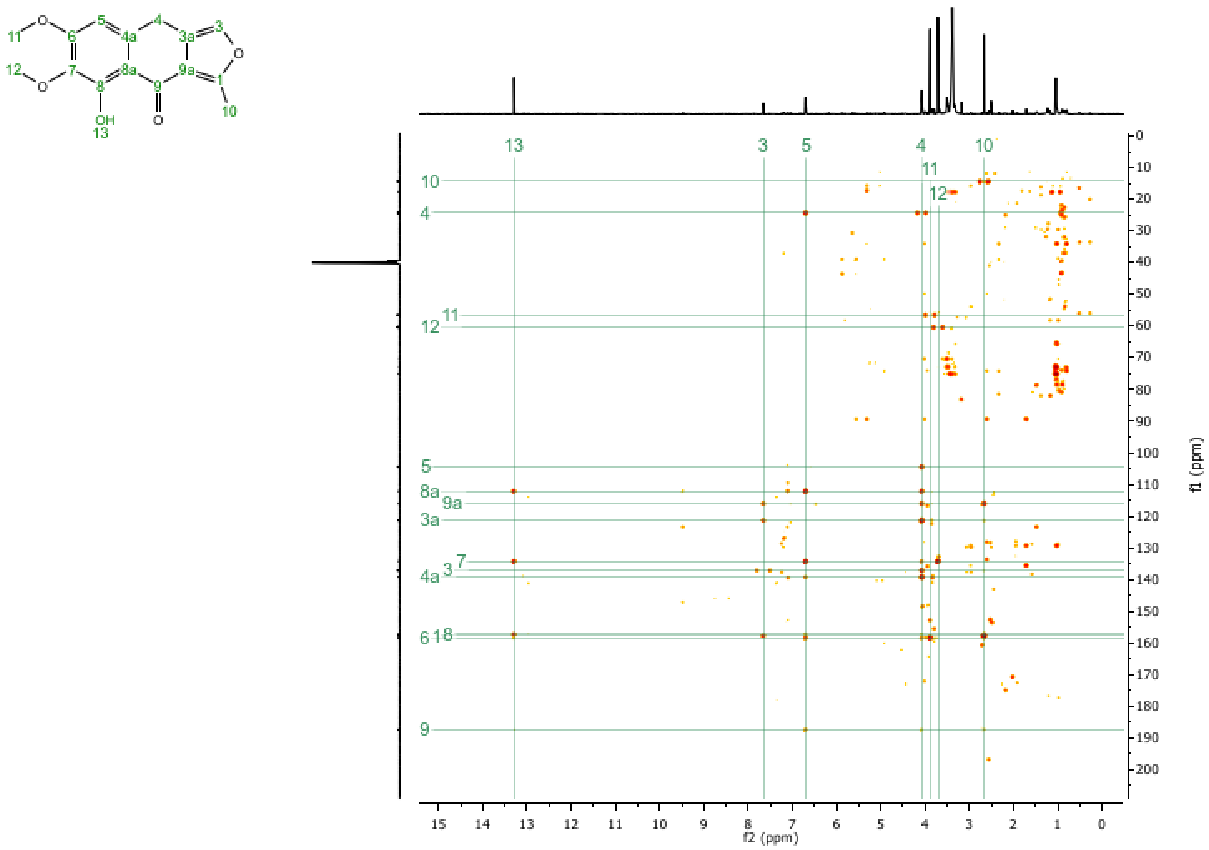

(g) ^{1}H-^{13}C HMBC NMR of NOC-IBR2

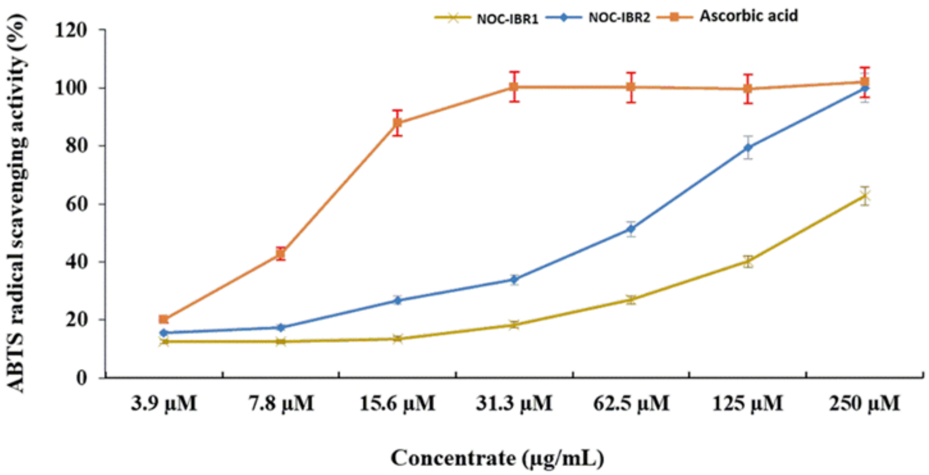

Supplementary Figure S5. The ABTS radical scavenging activity of NOC-IBR1, NOC-IBR2, and Ascorbic acid.

REFERENCES

Dhakal, D., Han, J. M., Mishra, R., Pandey, R. P., Kim, T.-S., Rayamajhi, V., et al. (2020). Characterization of Tailoring Steps of Nargenicin A1 Biosynthesis Reveals a Novel Analogue with Anticancer Activities. *ACS Chem. Biol.* 15, 1370–1380. doi: 10.1021/acschembio.9b01034

Dhakal, D., Kumar Jha, A., Pokhrel, A., Shrestha, A., and Sohng, J. K. (2016). Genetic Manipulation of Nocardia Species. *Curr. Protoc. Microbiol.* 40, 10F.2.1-10F.2.18. doi: 10.1002/9780471729259.mc10f02s40

Mishra, R., Dhakal, D., Han, J., Lim, H., Jung, H., Yamaguchi, T., et al. (2019). Production of a Novel Tetrahydroxynaphthalene (THN) Derivative from Nocardia sp. CS682 by Metabolic Engineering and Its Bioactivities. *Molecules* 24, 244. doi: 10.3390/molecules24020244

Sohng, J. K., Yamaguchi, T., Seong, C. N., Baik, K. S., Park, S. C., Lee, H. J., et al. (2008). Production, isolation and biological activity of nargenicin from Nocardia sp. CS682. *Arch. Pharm. Res.* 31, 1339–1345. doi: 10.1007/s12272-001-2115-0

Guo, Z., Pan, G., Xu, Z., Yang, D., Hindra, Zhu, X., et al. (2017). New isofuranonaphthoquinones and isoindolequinones from Streptomyces sp. CB01883. *J. Antibiot. (Tokyo)* 70, 414–422. doi: 10.1038/ja.2016.122

Katsuyama, Y., Sone, K., Satou, R., Izumikawa, M., Takagi, M., Fujie, M., et al. (2016). Involvement of the Baeyer-Villiger Monooxygenase IfnQ in the Biosynthesis of Isofuranonaphthoquinone Scaffold of JBIR-76 and -77. *ChemBioChem* 17, 1021–1028. doi: 10.1002/cbic.201600095
